# Oligomers Are Promising Targets for Drug Development in the Treatment of Proteinopathies

**DOI:** 10.3389/fnmol.2019.00319

**Published:** 2020-01-31

**Authors:** Oxana V. Galzitskaya

**Affiliations:** ^1^Laboratory of Bioinformatics and Proteomics, Institute of Protein Research, Russian Academy of Sciences, Pushchino, Russia; ^2^Laboratory of the Structure and Function of Muscle Proteins, Institute of Theoretical and Experimental Biophysics, Russian Academy of Sciences, Pushchino, Russia

**Keywords:** amyloid, oligomer, drug, polymorphism, model

## Abstract

Currently, there is no effective treatment of proteinopathies, as well as their diagnosis in the early stages of the disease until the first clinical symptoms appear. The proposed model of fibrillation of the Aβ peptide and its fragments not only describes molecular rearrangements, but also offers models of processes that occur during the formation of amyloid aggregates. Since this model is also characteristic of other proteins and peptides, a new potential target for drug development in the treatment of Alzheimer’s disease (AD) and other proteinopathies is proposed on the basis of this model. In our opinion, it is oligomers that are promising targets for innovative developments in the treatment of these diseases.

## Introduction

In the process of folding, the protein molecule acquires a unique spatial structure, which is necessary for its biological function. Nevertheless, in cells, there are a number of conditions under which the process of protein folding is disrupted. This leads to the formation of protein oligomers forming insoluble aggregates. A variety of such aggregates are amyloid fibrils. The formation and accumulation of amyloid aggregates in organs and tissues is one of the observed stages of the pathogenesis of diseases, combined into a group of proteinopathies, which includes Alzheimer’s disease (AD), Parkinson’s disease (PD), type 2 diabetes mellitus, and various systemic amyloidoses (Saha et al., 2000; Hardy and Selkoe, 2002; Caughey and Lansbury, 2003; Chiti and Dobson, 2006; Lesné et al., 2006; Shankar et al., 2008).

Currently, there is no effective therapy for proteinopathies, as well as their diagnosis in the early stages of the disease until the first clinical symptoms appear. In addition, a large number of proteins that are not associated with pathological processes are capable of forming amyloid aggregates and fibrils *in vitro*. This allows us to conclude that the formation of amyloids is a common property of the polypeptide chain (Fändrich and Dobson, 2002). It is also known that amyloid fibrils formed by the same protein can have a high degree of polymorphism (Fändrich et al., 2009). Therefore, the study of the molecular mechanism of the pathogenesis of amyloidosis is one of the urgent and important tasks of modern medicine and molecular biology.

## The Effectiveness of Drug Therapy

It is extremely alarming that the inefficiency of modern methods of treatment is associated with failures in the development of new drugs for the treatment of AD. The proportion of successful treatment attempts created by drugs during the decade from 2002 to 2012 is 0.4% (Ousset et al., 2014).

Cholinesterase Inhibitors (ChEIs) are a common form of drug treatment of AD, and the three most effective drugs are donepezil, galantamine, and rivastigmine. Side effects when using these drugs are different, but none of them contributes to a significant improvement in cognitive function in patients (Birks, 2006). There is evidence that prolonged exposure to these drugs even accelerates AD (Lu and Tune, 2003). In addition, they effectively increase the level of acetylcholine available for neurotransmission. Memantine is an alternative approved drug that only mildly inhibits the glutamatergic system by binding to N-methyl-D-aspartate receptors (NMDARs; Glasgow et al., 2017), which reduce excess Ca^2+^ in postsynaptic neurons associated with neurodegenerative diseases (Parsons et al., 2013). Glutamate receptors of the central nervous system play a key role in ensuring the plasticity of neurons and the processes of memory consolidation (under normal conditions). Hyperactivation of the N-methyl-D-aspartate (NMDA) subtype of these receptors leads to the development of neurotoxicity.

Memantine is also effective in combination with ChEIs (Tariot et al., 2004). Non-specific treatments for AD used include antidepressants, such as selective serotonin reuptake inhibitors fluoxetine and paroxetine, which can combine well with ChEI (Aboukhatwa et al., 2010). Other symptoms of AD, such as anxiety and psychosis, may be affected by drugs such as anxiolytics, oxazepam or antipsychotics, risperidone (Ballard and Waite, 2006). Although these drugs are considered effective in the treatment of AD, they nevertheless affect only the symptoms of the disease.

From the point of view of drug targets in the treatment of AD, α-, β- and γ-secretases are studied, which are involved in APP proteolysis to the Aβ peptide. As mentioned above, the disruption of the aggregation of the Aβ peptide can lead to the prevention of plaque formation (Yang et al., 2019). There are several targets associated with the degradation of the Aβ peptide, one of which is neprilysin (Hornung et al., 2019). There are targets that regulate the expression of APP in patients with AD. It is also necessary to include targets related to the phosphorylation and aggregation of tau protein in this incomplete list.

As for β-secretase (BACE1), there are many studies on its inhibition, including docking of a number of flavonoids (Shimmyo et al., 2008), as well as a number of studies on virtual screening (Huang et al., 2005; John et al., 2011); later high-throughput screening (in combination with pharmacophore modeling to clarify), which revealed the reasons for the inhibition of this enzyme (Muthusamy et al., 2013). Studies of mutant forms of BACE1 in mice indicate that there may be serious side effects when inhibiting this particular enzyme. In particular, such effects can be neurodegeneration, which is a serious problem (Yan and Vassar, 2014). As regards γ-secretase, this intramembrane protein is involved not only in the APP proteolysis but also in a number of other processes (Minter et al., 2005). It is clear that inhibition of this enzyme leads to a decrease in the amount of Aβ peptide (He et al., 2010). The situation is complicated by another protein, β-arrestin 2, which apparently regulates γ-secretase, and thus, inhibition of this enzyme can reduce the formation of Aβ peptide plaques (Thathiah et al., 2013). Since the formation of the Aβ peptide is a sequential process from the APP precursor protein that requires the sequential participation of BACE1 and γ-secretase, combination therapy, including both BACE1 inhibitor and γ-secretase modulator, will be more effective than an individual treatment of each individual enzyme during the formation of Aβ peptide (Strömberg et al., 2015).

## Amyloid Fibrils and Oligomers

There are two competing hypotheses about the cause of AD: one of them is the amyloid hypothesis (Tanzi and Bertram, 2005). It is based on the idea that the amyloid Aβ peptide, instead of being synthesized and participating in metabolism, begins to accumulate in the brain and form aggregates in the form of plaques. The accumulation of the peptide leads to pathology, expressed in the death of neuron cells and the appearance of plaques containing this protein. Genetic data are also a source of confirmation of this hypothesis. The Aβ peptide precursor protein (APP) gene is located on chromosome 21, and trisomy of this chromosome in Down syndrome is the reason that AD is often observed in patients with Down syndrome (Masters et al., 1985; Maltsev et al., 2011), which in this case indicates the genetic basis of AD disease. Simple proteolysis is required to convert the Aβ peptide precursor protein (APP) to the Aβ peptide. It should be mentioned that no correlation was found between amyloid plaque formation and neuronal loss (Schmitz et al., 2004). The so-called “channel” hypothesis of AD, first proposed in 1993, states: that oligomers of amyloidogenic proteins make pores into the membrane that causes the influx of Ca^2+^ ions, an imbalance of ions of other metals, oxidative stress, and finally cell death (Arispe et al., 1993). The second hypothesis is associated with modifications of tau protein. Hyperphosphorylation of tau protein associated with microtubules leads to pathology of neural tangles. Recent studies have shown that there is a connection between these two hypotheses (Small and Duff, 2008; Jin et al., 2011; Maltsev et al., 2014). In addition to this, misfolding of the Aβ peptide and tau protein is observed, which leads to their uncontrolled aggregation. Observation of the pathological process shows that misfolding is distributed from local points by the prion-like mechanism for both tau and Aβ peptide (Bloom, 2014). For tau protein, the formation of polymorphic particles was shown by NMR analysis (Mukrasch et al., 2009).

In the last decade, several researchers have attempted to describe oligomeric particles, which are possibly the precursors of the formation of amyloid fibrils. The direct interest in oligomeric particles is due to the fact that, for example, in the case of AD, oligomers formed by the Aβ peptide are found in the brain tissues of patients suffering from this disease (Roher et al., 1996). A similar role of oligomers (nanomers and dodecamers) was noted in many studies since such particles have the highest toxicity compared to dimers, trimers, and tetramers. More and more facts indicate that in the pathogenesis of neurodegenerative diseases, it is the oligomers, and not the mature fibrils, that pose the greatest danger. Thus, it has recently been demonstrated that oligomers formed by the Aβ(1–42) peptide have a damaging effect on the blood-brain barrier, thereby disrupting brain homeostasis (Brkic et al., 2015).

It is believed that amyloid fibrils are specifically ordered aggregates characterized by the presence of a secondary structure of a certain type—a cross-β structure, in which β-sheets are parallel to the axis of fibril (Makin and Serpell, 2005). In the formation of amyloid aggregates by globular proteins, only part of their amino acid sequence is involved in the formation of the cross-β structure. The introduction of amino acid substitutions into these regions (amyloidogenic region) may affect the ability of the protein to form amyloid fibrils. The determination of amyloidogenic regions is necessary to understand the mechanism of amyloid aggregation and the pathogenesis of neurodegenerative diseases (Selivanova et al., 2016c; Surin et al., 2016).

In addition, it currently remains difficult to establish the spatial structure of fibrillar aggregates due to the limited capabilities of individual physicochemical methods. For example, the structure of amyloid fibrils can be determined using the method of solid-state NMR spectroscopy, however, this method is very time-consuming and ambiguous in the interpretation of the obtained data. Also, despite the fact that amyloidogenic proteins and peptides are the objects of study by a large number of researchers, and the appearance of a large number of publications at the moment there is no general model that describes the molecular mechanism of the formation of amyloid aggregates and fibrils (Fändrich et al., 2011). Protofibrils are not yet available on images from a cryogenic sample, and therefore high-resolution cryo-EM reconstructions from fibrils represent the average values of multiple conformations of protofilaments (Gremer et al., 2017). In this case, averaging as high-resolution information of individual protofilaments, as well as conformational variability in flexible regions, are lost (Seuring et al., 2018).

Using bioinformatics approaches and modern physicochemical methods, we studied the formation of amyloid aggregates of the Aβ(1–40), Aβ(1–42) peptides and their fragments, and a model is proposed that describes the mechanism of the structural organization of amyloid fibrils (Suvorina et al., 2015; Selivanova et al., 2016a,b,c, 2018a,b; Galzitskaya and Selivanova, 2017; Galzitskaya et al., 2018a,b; Galzitskaya, 2019).

Based on the developed kinetic model of amyloid formation, the sizes of primary and secondary nucleus of fibril formation were calculated for two isoforms of the Aβ peptide (Dovidchenko et al., 2014, 2016). Thus, for the Aβ(1–40) peptide, it was found that the size of the primary nucleus is two monomers, and for Aβ(1–42) three monomers. In this case, the size of the secondary nucleus for the Aβ(1–40) peptide is one monomer, and for Aβ(1–42) it is two monomers. Based on the obtained data, a structural model of the primary oligomer underlying the dodecamer was proposed (Dovidchenko et al., 2016).

It was shown by electron microscopy that the key structural element, which is the building block for the formation of amyloid fibrils, is a ring-like oligomer consisting of approximately 12 monomers for Aβ(1–40) and Aβ(1–42) peptides. The diameter of such an oligomer is 8–9 nm, and the height about 3 nm. The inner diameter of the ring oligomer is 3–4 nm. Ring-like oligomers are stacked in a fibril on a ring-to-ring or more frequently ring-on-ring basis with a slight overlap. Oligomers of this type are observed in electron micrographs from the moment when the amyloid formation process begins, and their number gradually decreases. Oligomers interact with each other not only on a ring-to-ring basis but can also attached to the side surface of fibrils due to lateral interactions, thereby increasing its thickness.

We have proposed an oligomer structure for the Aβ peptide and its fragments, which are very different from each other. The Aβ peptide oligomer consists of three primary oligomers that form a ring-like structure in a cross-section. Twelve monomers (56 kDa) form a tubular cylinder with an internal diameter of about 3–4 nm, and the salt bridges stabilize this oligomer: Arg5-Glu22 is formed between the primary oligomers, and Asp23-Lys28 is formed inside the monomeric structure. The regular sizes of the structures observed upon application of these oligomers are present on X-ray diffraction patterns (this is the meridional reflection at 53Å and the equatorial reflection at 55Å). In the case of oligomer for the fragments of Aβ peptide, such an oligomer is formed from 48 peptides that form 12 β-sheets arranged in a tubular cylinder with outer and inner diameters of 6 and 2 nm, respectively (Galzitskaya et al., 2018b).

**Figure 1:**
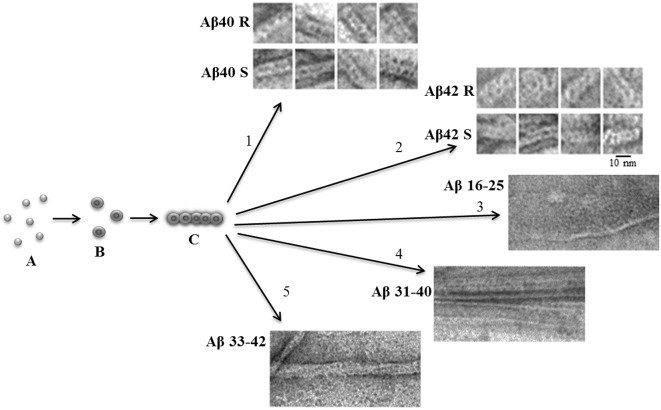
Schematic representation of the possible mechanism of fibril formation by Aβ peptide and its fragments. **(A)** Monomers; **(B)** ring-like oligomers; and **(C)** single fibril. R is a recombinant sample and S is synthetic.

An important characteristic of amyloid fibrils formed by Aβ(1–40) and Aβ(1–42) peptides is their polymorphism. The model of aggregation of amyloid fibrils that we have proposed is valuable in that it explains this polymorphism. In this case, this is due to a change in the association of oligomers during amyloidogenesis (Figure 1). The structure of the oligomer itself is determined by the amino acid sequence of the monomers. A vivid example of this can be demonstrated by the example of Aβ(1–40) and Aβ(1–42) isoforms and amyloidogenic fragments of the Aβ peptide. The constructions of oligomers differ in the structure of the complex and physicochemical properties (Dovidchenko et al., 2014; Galzitskaya and Selivanova, 2017; Selivanova et al., 2018b; Galzitskaya, 2019; Figure 1).

To determine the amyloidogenic regions of the protein polypeptide chain that form intermolecular interactions in amyloid fibrils, an approach was used consisting in the limited proteolysis of mature fibrils and subsequent determination of the amino acid sequence of the obtained peptides using high-resolution mass spectrometry (Selivanova et al., 2016c; Surin et al., 2016; Galzitskaya et al., 2018b).

The data obtained using the approach described above allowed us not only to find out what structural transformations a protein or peptide molecule undergoes in the process of fibril formation, but also to confirm the described model of amyloid formation for Aβ(1–40) and Aβ(1–42) peptides. It should be noted that the experimentally determined amyloidogenic fragments coincided with the predicted sites using the FoldAmyloid program (Garbuzynskiy et al., 2010).

## Why Do Not Mice Get Alzheimer’s Disease? A Possible Organization of The Oligomeric Structure May Provide An Answer to This Question

Recent evidence suggests that soluble Aβ peptide oligomers are a major cause of synaptic dysfunction and memory loss in AD. To further address this uncertainty, the neurotoxicity of various isoforms of Aβ peptide was analyzed at the cellular level. The results showed that Aβ(1–42) can form oligomers much faster than Aβ(1–40) oligomers, while Aβ(1–43) and Aβ(1–42) exhibit the highest level of neurotoxicity (Fu et al., 2017).

The EM images clearly show that fibrils are built from oligomeric structures for both the Aβ peptide and its fragments (Figure 1). The structure of Aβ(1–42) fibril, determined using cryo-electron microscopy, does not coincide with the EM images presented in the Supplementary: when increasing the EM images, it is clear that the fibril does not consist of endless beta-sheets obtained using the processing program cryo-EM, and the fibril is constructed of oligomeric structures laid in the same way as in our model (Gremer et al., 2017).

**Figure 2:**
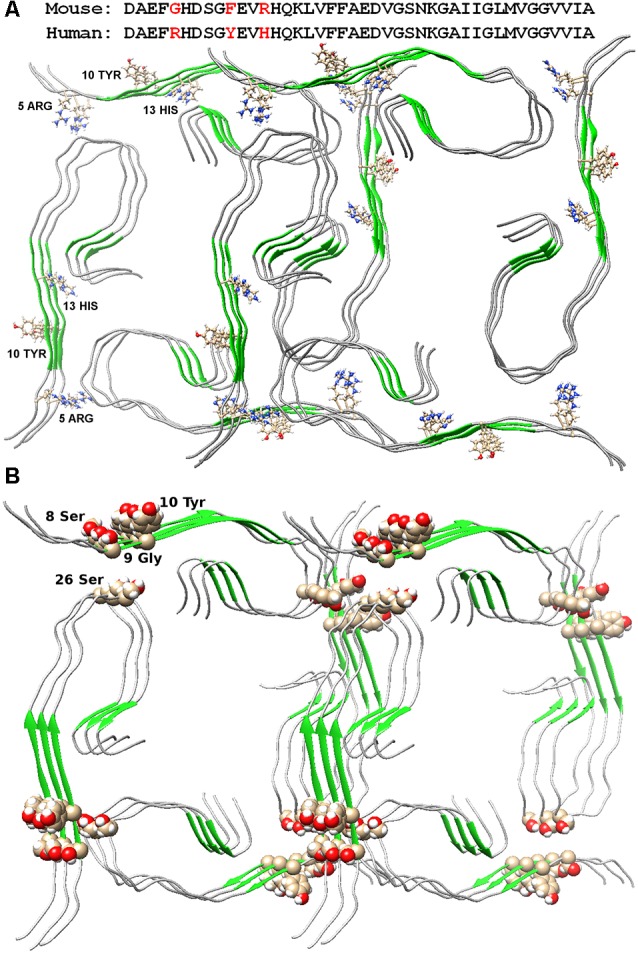
Packing of two dodecamer structures of Aβ(1–42) peptide in fibril. **(A)** Amino acid sequences of mouse and human Aβ(1–42) peptide are presented. Arg5, Tyr10, and His13 are indicated in the structures. **(B)** Ser26 and nearby residues are highlighted in the structure.

The possible organization of the oligomeric structure may answer the question, why mice do not have AD? In the mouse Aβ peptide, Gly is located instead of Arg5, thereby violating the salt bridge, a bond that stabilizes the layers of primary oligomers. And the presence of Arg13 instead of His13 only prevents the formation of such an oligomer structure. Thus, the replacement of three amino acids at the N-terminus of the murine Aβ peptide results in no signs of AD in mice (Figure 2A). The deletion of Glu22 (Osaka mutant) causes enhanced oligomerization of the Aβ peptide, but not fibrillogenesis (Tomiyama et al., 2008). Again, a salt bridge cannot form, a bond stabilizing the monomeric form of the Aβ peptide. Ala2Thr mutation (Jonsson et al., 2012) can slow down Aβ fibrillogenesis (Lin et al., 2017), but at the same time, Ala2Val mutation (Di Fede et al., 2009) accelerates Aβ fibrillogenesis and is associated with the early onset of AD (Messa et al., 2014), since such mutation leads to stabilization of the N-terminal part of the peptide. English mutation (Janssen et al., 2003) His6Arg promotes fibrillogenesis, enhances cytotoxicity, and increases the average size of Aβ oligomers (Ono et al., 2010). It should be noted that for only a few mutants, the model structure of monomer packing in amyloid fibril was obtained.

Five developed antibodies (Gantenerumab, Solanezumab, Aducanumab, Bapineuzumab, Crenezumab) did not reach clinical stage 3. This means that there was a misconception (vision) of the structure of the amyloid against which antibodies were developed. Among the ensemble of oligomers, it is necessary to single out the “correct” oligomer, which is involved in the construction of fibrils. And as we now understand, such an oligomer should be just 56 kDa dodecamer. It is just stable compared with the primary oligomer—tetramer. Murakami (2014) presents in his article a picture with a large pool of oligomers that will participate in the construction of fibrils. But if we take into account that the fibril is built from specific building material, then all the other oligomers should not interest us as a target.

The Ser26Glu mutation was detected in the Aβ peptide (a message from S. Linse at the Amyloid 2019 conference in Lund), which does not lead to cross-seeding, which means that the structures from which the fibrils are built for the wild type and mutant shape are different (Tran et al., 2017). From the point of view of existing structures, such a mutation should not affect the fibril structure in any way, since in both structures of 2016 and 2017, this residue looks at the solvent (Wälti et al., 2016; Gremer et al., 2017). Only in our model, this mutation will prevent the formation of an oligomeric particle, and most likely, the mutant form will have a completely different structure of the building block—the oligomer, so the process of cross-seeding is impossible (Figure 2B).

## Conclusions

The study of the reasons and development of neurodegenerative diseases is an important and urgent task of modern medicine. The prevalence of these diseases is from 5 to 15%, depending on the age of the patient. It should also be noted that the spread of this group of diseases is also an acute social problem, since these diseases reduce the quality and life of patients. As a rule, such diseases are diagnosed in the late stages of development, when patients develop the impaired cognitive function. At the moment, the etiology and pathogenesis of various neurodegenerative diseases and proteinopathies are only being clarified, there are almost no diagnostic methods at an early stage of the disease and attempts to develop an effective method of treatment have practically no results. This is a direct consequence of a lack of understanding of key events in the molecular mechanism of the pathogenesis of neurodegenerative diseases and proteinopathies.

Our proposed model of fibrillation of Aβ peptide and its fragments not only describes molecular rearrangements, but also offers models of processes that occur during the formation of amyloid aggregates. In addition, we offer a new potential target for drug development in the treatment of AD. In our opinion, it is “correct” oligomeric complexes that are promising targets for innovative developments in the treatment of this disease.

## Data Availability Statement

The datasets generated for this study are available on request to the corresponding author.

## Author Contributions

The author confirms being the sole contributor of this work and has approved it for publication.

## Conflict of Interest

The author declares that the research was conducted in the absence of any commercial or financial relationships that could be construed as a potential conflict of interest.
